# Erectile dysfunction among diabetic patients in Western Uganda: prevalence and associated factors in a multicentre study across three selected clinics

**DOI:** 10.1186/s12902-025-02048-2

**Published:** 2025-10-23

**Authors:** Venance Emmanuel Mswelo, Afizi Kibuuka, Tijjani Salihu Shinkafi, Wardat Rashid Ali, Elias Joseph Xwatsal, Abdisamad Guled Hersi, Zakarie Abdullahi Hussein, David Elia Saria, Wisdom Njumwa, Hanan Asad Hassan, Abukar Ali Ahmed, Adan Abdi Hassan, Abdisalam Ahmed Sandeyl, Theoneste Hakizimana, David Mumbere Mayani, Josiah J. Mkojera, Feisal Dahir Kahie, Dalton Kambale Munyambalu, Jacinto Amandua

**Affiliations:** 1 Department of Internal Medicine, Faculty of Clinical Medicine and Dentistry, Kampala International University, Kampala, Uganda; 2 Department of Internal Medicine, Jinja Regional Referral Hospital, Jinja, Uganda; 3 Department of Biochemistry, Kampala International University, Kampala, Uganda; 4 Department of Public Health and Nutrition, Faculty of Health Sciences, Victoria University, Kampala, Uganda; 5 Department of Obstetrics and Gynaecology, Faculty of Clinical Medicine and Dentistry, Kampala International University, Kampala, Uganda; 6 Medical Department, Ligula Regional Referral Hospital, Mtwara, Tanzania

**Keywords:** Prevalence, Erectile dysfunction, Diabetes mellitus, Multicentre, Clinics, Hoima, Fort portal, KIU, Western Uganda

## Abstract

**Background:**

Erectile dysfunction (ED) is the inability to achieve or sustain an erection adequate for satisfactory sexual activity. The prevalence of ED varies widely among diabetic patients worldwide, mainly due to disparities in healthcare access and diabetes management between low-income and high-income countries. ED significantly impacts the quality of life for affected men, potentially leading to frustration, despair, and sometimes separation from an intimate partner. This study aimed to assess the prevalence, severity patterns, and factors associated with erectile dysfunction among diabetic men attending clinics in three selected sites in Western Uganda.

**Methods:**

The clinic-based cross-sectional study was conducted involving 236 diabetic men from three clinics: Fort Portal Regional Referral Hospital, Hoima Regional Referral Hospital, and Kampala International University (KIU) Teaching Hospital in Western Uganda from June to September 2024. The number of patients was proportionally assigned depending on the number of diabetic men receiving care at the selected facilities. For each clinic, the patients were enrolled consecutively. The IIEF-5 questionnaire was used. Descriptive statistics and bivariate and multivariable logistic regression were done with SPSS version 20.0.

**Result:**

The prevalence of erectile dysfunction was 79.2%, whereby 21.95%, 36.4%, 22.4% and 19.3% had mild, mild to moderate, moderate, and severe erectile dysfunction, respectively, at 95% CI. Multivariable logistic regression identified increasing age, HbA1c (aOR = 2.44, 95%CI:1.067–5.569, *p* = 0.035), fasting blood sugar (aOR = 2.50, 95%CI:1.106–5.657, *p* = 0.028), and BMI (aOR = 15.1833, 95%CI: 1.168–19.739) to be significantly associated with ED. No behavioural characteristic was associated with ED.

**Conclusion:**

The prevalence of erectile dysfunction was very high. Increasing age, obesity, and inadequate control of blood sugar were significantly associated with ED. Future studies should focus on qualitative data and causal and therapeutic interventions. We also recommend routine screening and timely addressing of blood sugars.

**Supplementary Information:**

The online version contains supplementary material available at 10.1186/s12902-025-02048-2.

## Background

The National Institute of Health (NIH) Consensus and the American Urological Association define erectile dysfunction as a man’s incapacity to achieve and sustain a penile erection strong enough to allow for fulfilling sexual activity [1, 2].

The 2024 United Nations (UN) and World Health Organisation (WHO) reports estimated that about 828 million adults (18 years and above) have diabetes globally. The prevalence is soaring in low-income and middle-income countries seems higher while are burdened with poor treatment coverage [3–5]. In Uganda, the prevalence of diabetes in adults is 3.6% with the total number of cases of diabetes in adults being about 716,000 [6–8].

Erectile dysfunction (ED) is one of the most prevalent DM complications yet overlooked. Its global prevalence varies with places and methodological approaches, but is generally estimated to range from 35 to 94.7% [9, 10] for lower and higher-income countries, respectively. The lower prevalence has been reported in the American region, while higher figures have been reported in the European and the African regions [11, 12]. Erectile dysfunction poses a threat to the quality of life of affected men [10, 13]. ED can cause frustration, despair, and sometimes separation from intimate partners [14].

ED is associated with many factors, either unifactorial or multifactorial, coming into conjunction in a diabetic patient. Several phenomena like vasculogenic, neurologic, or psychologic as one entity or in combination may play a core culprit in ED pathogenesis [11, 15, 16].

Socio-demographic factors like age [9, 11, 17] Marital Status and Occupation [9] are independently associated with ED. Behavioural factors such as alcohol consumption and smoking have shown non-uniform findings in different studies [18–21]. Physical exercise was a protective factor in some studies [20, 22] while some activities, like riding a bicycle, may cause sexual dysfunction by exerting direct pressure on the scrotum [23].

Medical factors that may influence ED in patients include: peripheral neuropathy [11, 15]., Hypertension [11, 15] Body mass index [11, 24, 25] Duration of Diabetes mellitus [9, 11] [20, 26]., glycemic control, and drug adherence [9, 11, 15, 20, 25].

The WHO statement in 2017 recognises that normal erectile function stands to be an essential part of the sexual and reproductive health of men, and therefore, addressing any form of sexual dysfunction is part of improving health [27].

ED may sabotage marital and relationship issues, cultural norms and expectations, individual self-esteem, dignity, and precipitate anxiety and depression. ED may cause significant emotional damage to the patient and/or their spouse, subsequently harming their quality of life. Up to 3% of patients using phosphodiesterase-5 inhibitors for ED may succumb to priapism [23].

The data regarding erectile dysfunction among men living with diabetes in Uganda are scarce. Therefore, this multi-centre study explored the recent magnitude of erectile dysfunction as well as addressed its determinants among diabetics.

## Materials and methods

### Study design and setting

A clinic-based cross-sectional study was conducted at three health facilities: Kampala International University Teaching Hospital (KIUTH), Fort Portal Regional Referral Hospital (FPRRH), and Hoima Regional Referral Hospital (HRRH) from 10th June to 9th September 2024.

KIU-TH is located in Ishaka-Bushenyi municipality, Western Uganda. It is the largest private teaching hospital in Uganda. It is a non-profit hospital situated about 5 km away from Bushenyi district headquarters. KIU-TH serves as a referral centre for these neighbouring hospitals of Ishaka Adventist, Comboni, and Kitagata. Patients visit the medical outpatient clinic (MOPD) daily on weekdays. The clinic serves between 70 and 100 male DM patients.

Fort Portal Regional Referral Hospital is located within the city of Fort Portal, approximately 294km west of Mulago National Referral Hospital, in Kampala. It is a public hospital with over 300 patient beds. It serves several districts of Bundibugyo, Kabarole, Kamwenge, Kasese, Ntoroko, and Kyenjojo. The diabetic outpatient clinic is open every Thursday. The clinic serves about 150–200 male diabetes patients as per a September 2023 unpublished survey.

Hoima Regional Referral Hospital, commonly known as Hoima Hospital, is the main hospital in Hoima city. It is located 200km west of Kampala. It serves as a referral facility for several districts of Bulisa, Hoima, Kibaale, Kiryandongo, Kagadi, Kakumiro, Kikuube, and Masindi, and so overall grossing over 3 million people. The diabetic outpatient clinic is open on Wednesdays. The clinic also serves about 150–200 male diabetes patients, as per the hospital’s unpublished data.

### Source and study population

#### Source population

The source population included all adult diabetic male patients attending clinic follow-ups at KIUTH, FPRRH, and HRRH during the study period.

#### Study population

All diabetic patients who were 18 years of age or older and who consented to participate in the study visited the diabetic clinics (KIU-TH, FPRRH, and HRRH) during the study’s duration.

#### Inclusion criteria

Males aged 18 years and above were diagnosed with diabetes mellitus at least 6 months before the commencement date of the study.

#### Exclusion criteria

Any diagnosed mental disorders or inability to stand an interview or dissented any time during the study, Patients with a diagnosis of ED before the diagnosis of DM, those who were celibates (for non-ED reasons), known secondary ED from genetic, endocrine, neurological (multiple sclerosis, stroke), or surgical causes, patients with a history of major pelvic surgeries (example prostatectomy, symphysiotomy and so on).

### Sample size determination

Sample size was calculated using Cochran’s (1963:75) formula.

$$n_0=\;\frac{Z^2pq}{e^2}$$

nₒ = desired sample size for a large population greater than 10,000.

Z² = standard normal deviation, assuming a 95% confidence interval, 1.96.

P = proportion in the population estimated to have ED = 55.1% from Tanzania [15].

q = (1-P) = (1-0.551) = 0.449.

*ℯ *= is the desired level of precision for a 95% confidence interval (0.05).

nₒ = (1.96 ²×0.449 × 0.551)/(0.05)².

nₒ = 0.77107/0.0025.

nₒ = 380 participants.

For a finite population, the sample size is then corrected further.

$$n=\frac{n_0}{1\;+{\displaystyle\frac{\left(n_0-\;1\right)}N}}$$

Where “n” is the sample size and “N” is the population size (500 men), *n* = 380 ÷ [1 + (380-1)/500] = 216.

Considering a 10% attrition factor, the estimated sample size was about 236 participants.

Diabetic clinics at KIUTH, FPRRH, and HRRH serve between 370 and 500 diabetic male patients in total, implying we can reach the required sample size. Given the population distribution among the three hospitals, a ratioed 236 participants were enrolled during the duration of our research; 48, 94, and 94 from the three respective sites.

### Sampling techniques

The three hospitals were selected purposely to represent the community in the Western region. At each clinic, the study participants were enrolled consecutively until the desired sample size was reached.

### Data collection tools

Data was collected using a questionnaire presented by the interviewer that was written in English (later translated to local languages, Runyankore, Rutooro, and Runyoro) based on the facility, and most importantly problem statement and objectives. The International Index of Erectile Function-5 (IIEF-5) is a widely used questionnaire in the assessment of ED. It has been recommended by the American Society of Urology and has a sensitivity of 98% and a specificity of 88% [28] The questionnaire has scores ranging from 5 to 25 where: A score of 22–25 for normal erectile function, 17–21 for Mild erectile dysfunction, 12–16 for Mild to moderate erectile dysfunction, 8–11 for Moderate erectile dysfunction, 5–7 for severe erectile dysfunction. The study questionnaire was divided into multiple sections to include preliminary biodata information, associated factors for diabetic erectile dysfunction (including AUDIT score for alcohol intake, anthropometry, laboratory parameters) and the erectile function Index score. Assessment of physical exercise was also done subjectively by asking the participant if they do exercise with a No/Yes response tallied to minutes per day and frequency per week.

### Data collection procedure and recruitment

Data was collected by research assistants who were residents of Internal Medicine at respective hospitals. The process was well organised and supervised by the principal investigator.

#### Preliminary procedure

Participants were made aware of the study (and role of blood sample collected) and asked to sign a written consent form. Following that, information on sociodemographic traits (age, sex, marital status, address, levels of education), behavioural traits (alcohol usage, cigarette smoking), and medical histories were filled in.

#### Anthropometry and blood pressure

Weight in Kilograms measured on a given day of the follow-up was recorded from the patient’s charts. Height measured to the closest 0.1 centimetre on the same day was also recorded. The patient’s weight in kilograms divided by their height in meters squared yielded BMI. High blood pressure was defined as a diastolic blood pressure of 90 or a systolic blood pressure of 140 or higher [29].

#### Examination of peripheral neuropathy

A physical assessment for peripheral neuropathy was done. Pressure sensation was assessed using a 10 g monofilament (Semmes-Weinstein test). Patients were asked to close their eyes while the monofilament was pressed perpendicular at 9 areas of the plantar surface and one on the dorsum of the foot until it buckled. Saying “Yes” will imply that the patient can feel the monofilament every time it is pressed. Failure to feel the microfilament 4 out of 10 times of pressing indicates peripheral neuropathy. The test has a sensitivity of 97% and a specificity of 83% for detecting peripheral neuropathy [15].

#### Assessment of erectile dysfunction

Erectile Function was assessed by the International Index of Erectile Function Questionnaire (IIEF-5). The tool was adopted from the American Urological Association in −2018. It consists of five questions which assess the individual’s erectile functioning and ability to sustain intercourse. The scores of 5–7, 8–11, 12–16, 17–21 and 22–25 represented severe, moderate, mild to moderate, and no erectile dysfunction, respectively.

#### Laboratory parameters

The capillary fasting blood glucose (FBS) measured on a given day was collected from the patient’s chart. Three millilitres (3 ml) of venous blood were collected into a test tube containing EDTA (ethylene diamine tetra-acetic acid) to measure glycosylated haemoglobin (HbA1c), which was done at KIUTH’s main laboratory. A triple packaging system was used to transport the samples from FPRRH and HRRH to KIUTH. The samples were maintained in the cooler box at a temperature of 2 to 8 °C. The transportation cooler box was sanitised before and after transport.

### Study variables

Independent variables included: socio-demographic factors, behavioural characteristics (smoking, alcohol use, physical exercise), and medical characteristics (glycaemic control, comorbidities, duration of DM). Erectile dysfunction (IIEF-5 score) was the studied dependent variable.

### Quality control

Data was collected using a locally pretested questionnaire. Checking every day for completeness and accuracy of filled questionnaires was timely. A one-day training was done for data collectors by the principal investigator before data collection. Data cleaning was also done before data analysis. 10 randomly selected blood samples were taken to the Lancet laboratory in Ishaka for the HbA1c test as a part of external validity.

### Data management and analysis

Coded data was entered into Microsoft Excel 2019 and transferred into IBM SPSS version 20.0. Descriptive statistics were done to compute the baseline characteristics, prevalence, and severity pattern of erectile dysfunction (ED). Logistic regression was done to determine factors associated with ED. Variables with *p-value ≤ 0.2* bivariate level were taken into a multivariable logistic regression to adjust for possible confounders, with which a *p-value* of *< 0.05* was considered statistically significant.

### Ethical consideration

This work obtained ethical approval from Kampala International University REC with a registration number of KIU-2024-311. Participants’ informed consent was sought after a thorough explanation of the study’s specifics in English and/or Rutooro/Runyoro/Runyankore was given.

## Results

### Study profile

**Fig. 1:**
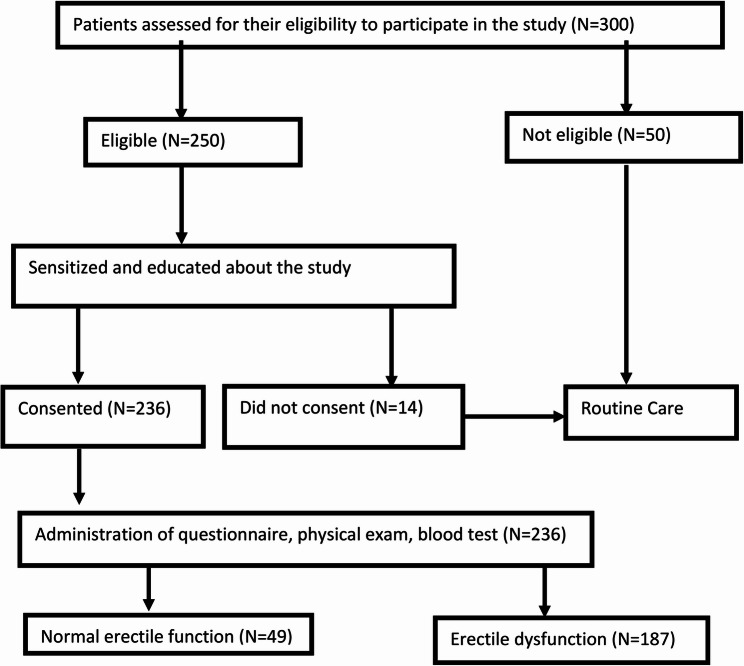
Study participants profile

From June to September 2024, 300 adult men with diabetes mellitus were assessed to determine if they met eligibility criteria to participate in this study. 50 men were excluded because 45 were celibates and 5 had erectile dysfunction before they were diagnosed to have diabetes mellitus. They therefore continued with routine care. Among 250 who were eligible for the study, 14 did not consent to the study because they just did not want to participate. The questionnaire, physical examination, and blood tests for HbA1c were feasible for the remainder of 236 (Fig. 1).

### Baseline characteristics of the study participants

The participants from three different clinics: 94, 94, and 48 participants from FPRRH, HRRH, and KIUTH, respectively. Most of the participants were from tribes of Tooro (31.8%), Nyoro (30.9% and Nyankole (17.8%). The mean age of the participants was 55 years. More than three-quarters had at least primary education, and about 61.9% were peasants. A total of 3 students were included and grouped under the unemployed occupation category (Table 1).

**Table 1:** The baseline characteristics of the study participants

Characteristics	Frequency (*N*)	Percent (%)
Heath Facility		
KIUTH	48	20.4
FPRRH	94	39.8
HRRH	94	39.8
Age (Years)		
Less than 30	15	6.3
30 to 39	20	8.5
40 to 49	41	17.4
50 to 59	58	24.6
60 and above	102	43.2
Marital Status		
Married/Cohabiting	212	89.8
Single/separated	24	10.2
Education		
Informal	49	20.8
Primary	107	45.3
Secondary	51	21.6
Tertiary	29	12.3
Occupation		
Unemployed	7	3.0
Business	54	22.9
Peasants	146	61.9
Professional	29	12.3
Tribe		
Tooro	75	31.8
Nyoro	73	30.9
Nyankole	42	17.8
Others	46	19.5
Subregion		
Ankole	48	20.3
Rwenzori	94	39.8
Bunyoro	94	39.8

### Behavioral characteristics of study participants

A minority of our study participants were either cigarette smokers or alcohol consumers, 24.2% and 20.8% respectively. 67.8% of study participants reported doing exercises regularly (Table 2).

**Table 2:** Behavioral characteristics of the study participants

Variable	Frequency	Per cent
Smoking		
Yes	57	24.2
No	179	75.8
Alcohol use		
Not taking alcohol	187	79.2
Low risk	31	13.1
Medium risk	17	7.2
Alcohol addiction	1	0.4
Exercise		
Yes	160	67.8
No	76	32.2

### Medical profile of the study participants

Of 236 study participants, 68.2% had diabetes for over five years. 54.6% were on oral hypoglycaemic medications alone, while 29.7% were on oral medications and insulin injections. About three-quarters had inadequate glycemic control (high HbA1c). Hypertension was the commonest comorbidity, constituting about 52.9%. Loss of protective sensation with a 10-g Semmes-Weinstein monofilament was noted in 23.3% of the study participants. 5.5% had underweight, 58.9% had a normal BMI (Table 3).

**Table 3:** The medical characteristics of the study participants

Variable	Frequency	Per cent
DM Duration		
less than 5 years	75	31.8
5 to 10years	70	29.7
more than 10 years	91	38.5
DM therapy		
Oral	129	54.6
Insulin	37	15.7
Insulin + Orals	70	29.7
HbA1c (%)		
Normal (≤ 7%)	62	26.3
Abnormal (>7%)	174	73.7
FBS (mmol/L)		
Normal (≤ 7)	72	30.5
Abnormal (>7)	164	69.5
Comorbid Conditions		
None	104	44.1
HIV	6	2.5
Hypertension	115	48.7
TB	1	0.4
TB/Hypertension	3	1.3
HIV/Hypertension	6	2.5
HIV/TB/Hypertension	1	0.4
Hypertension		
Yes	125	52.9
No	111	51.3
Peripheral Neuropathy (SW monofilament test)		
Abnormal	55	23.3
Normal	181	76.7
BMI(Kg/M^2^)		
Underweight (< 18.5)	13	5.5
Normal (18.5–24.9)	139	58.9
Overweight (25-29.9)	67	28.4
Obese (30+)	17	7.2

### Erectile function of participants

**Fig. 2:**
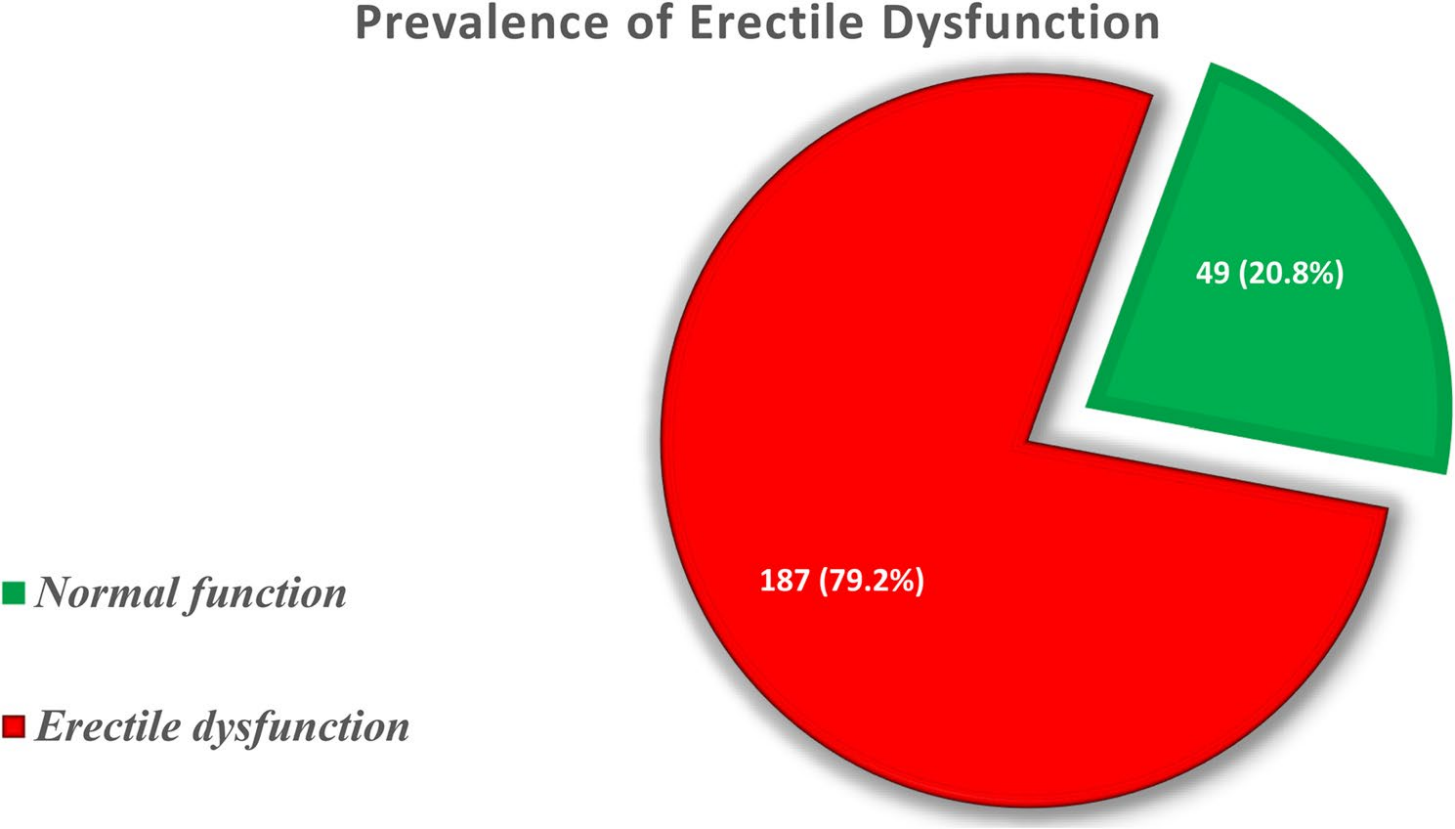
A chart showing the proportion of erectile dysfunction among male diabetic men attending the three clinics: KIUTH, FPRRH, and HRRH

Out of 236 adult diabetic men, 187 (79.2%,95% CI) were identified to have erectile dysfunction, with the remainder, only one-fifth, having a normal erectile function (Fig. 2).

### Pattern of erectile dysfunction

**Fig. 3:**
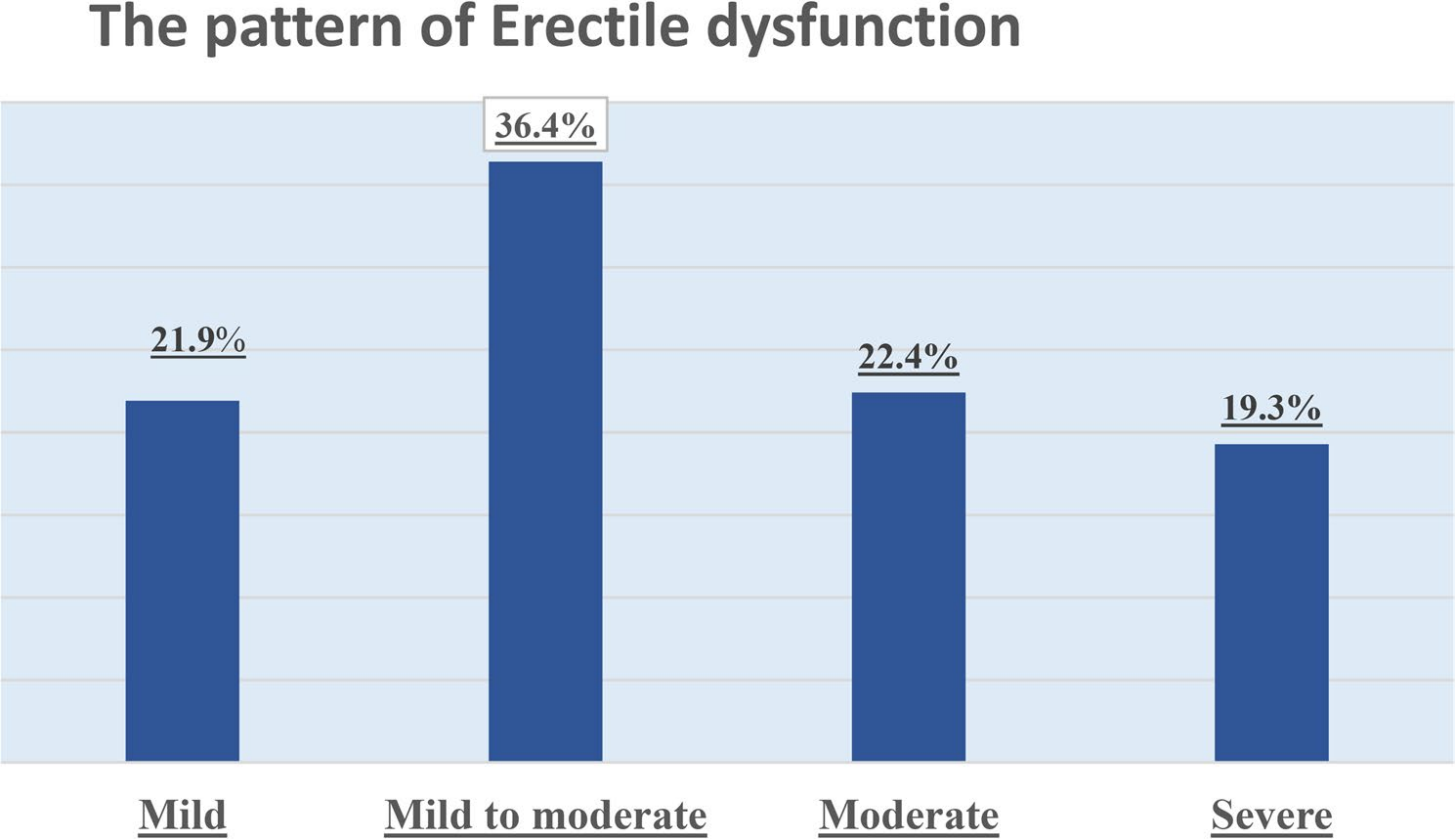
A chart showing the severity pattern of erectile dysfunction

The prevalence of erectile dysfunction was generally different from one hospital to another. The figures were 81.3%, 81.9% and 75.5% for KIUTH, FPRRH, and HRRH, respectively. Moreover, the severity patterns showed variable distribution in the study centres. The majority of men had mild to moderate (36.4%) and moderate (22.4%) erectile dysfunction (Fig. 3; Table 4).

**Table 4:** Distribution of erectile dysfunction at the three diabetic clinics: KIUTH, FPRRH, and HRRH

		Pattern of Erectile function					Total
		Normal	Mild	Mild to moderate	Moderate	Severe	
Health facility	KIUTH	9 (18.8%)	12 (25%)	10 (20.8%)	10 (20.8%)	7 (14.6%)	48 (100%0
	FPRRH	17 (18.1%)	18 (19.1%)	30 (31.9%)	16 (17%)	13 (13.8%)	94 (100%)
	HRRH	23 (24.5%)	11 (11.7%)	28 (29.8%)	16 (17%)	16 (17%)	94 (100%)
Total		49 (20.8%)	41 (17.4%)	68 (28.8%)	42 (17.8%)	36 (15.3%)	236 (100%)

### Factors associated with erectile dysfunction among adult diabetic men attending diabetic clinics at three selected hospitals

In bivariate analysis: age, marital status, fasting blood glucose, HbA1c, DM therapy, Hypertension, history of smoking, and monofilament test showed significant statistical association (*p* ˂ 0.05) with the presence of erectile dysfunction (Table 4). All variables with a *p-value* of ≤ 0.2 were entered into a multivariable model for analysis (Table 6). 

**Table 5:** Bivariate analysis of the factors associated with erectile dysfunction among diabetic men attending a clinic at KIUTH, FPRRH, and HRRH

Variable	Erectile Dysfunction		cOR (95% CI)	*p*-value
	Yes	No		
**Health Facility**				
KIUTH	39 (81.2%)	9 (18.8)	1.4 (0.592–3.331)	0.442
FPRRH	77 (81.9%)	17 (18.1%)	1.47 (0.725–2.969)	0.286
HRRH	71 (75.5%)	23 (24.5%)	1	
**Residence**				
Rural	140 (80%)	35 (20%)	1	
Urban	47 (77%)	14 (23%)	0.84 (0.4161.694)	0.625
**Age**				
Less than 30	7 (46.7%)	8 (53.3%)	1	
30 to 39	9 (45%)	11 (55%)	0.935 (0.244–3.584)	0.922
40 to 49	29 (70.7%)	12 (29.3%)	2.762 (0.818–9.329)	0.102*
50 to 59	51 (87.9%)	7 (12.1%)	8.327 (2.302–30.119)	0.001*
60 and above	91 (89.2%)	11 (10.8%)	9.455 (2.871–31.134)	0.000*
**Education level**				
Informal	43 (87.8%)	6 (12.2%)	1	
Primary	87 (81.3%)	20 (18.7%)	2.28 (0.683–7.612)	0.180*
Secondary	35 (68.6%)	16 (31.4%)	1.38 (0.520–3.686)	0.515
Tertiary	22 (75.9%)	7 (24.1%)	0.7 (0.247–1.961)	0.493
**Employment/Occupation**				
Professional	23 (79.3%)	6 (20.7%)	1	
Unemployed	3 (75%)	1 (25%)	0.78 (0.069–8.934)	0.844
Business	43 (79.6%)	11 (20.4%)	1.02 (0.334–3.113)	0.973
Peasants	116 (79.5%)	30 (20.5%)	1.01 (0.377–2.699)	0.986
Students	2 (66.7%)	1 (33.3%)	0.52 (0.040–6.770)	0.619
**Marital status**				
Single/separated	15 (62.5%)	9 (37.5%)	1	
Married/Cohabiting	172 (81.1%)	40 (18.9%)	2.580 (1.054–5.315)	0.038*
**DM duration**				
less than 5 years	58 (77.3%)	17 (22.7%)	1	
5 to 10years	51 (72.9%)	19 (27.1%)	0.79 (0.370–1.674)	0.533
more than 10 years	78 (85.7%)	13 (14.3%)	1.76(0.792–3.906)	0.166*
**Fasting blood sugar**				
Normal (≤ 7%)	48 (66.7%)	24 (33.3%)	1	
Abnormal (>7%)	139 (84.8%)	25 (15.2%)	2.78 (1.453–5.321)	0.002*
**HbA1c (%)**				
Normal (≤ 7%)	42 (67.7%	20 (32.3%)	1	
Abnormal (>7%)	145 (83.3%)	29 (16.7%)	2.38 (1.224–4.631)	0.009*
**Comorbid conditions**				
none	73 (70.2%)	31 (29.8%)	1	
HIV	4 (66.7%)	2 (33.3%)	0.849(0.1484.881)	0.855
Hypertension	102 (88.7%)	13 (11.3%)	3.332(1.6326.804)	0.001*
HIV/Hypertension	4 (66.7%)	2 (33.3%)	0.85 (0.148–4.881)	0.855
TB	0	1		
TB/Hypertension	3	0		
HIV/TB/Hypertension	1	0		
**Hypertension**				
No	110 (58.8%)	77 (41.2%)	1	
Yes	15 (30.6%)	34 (69.4%)	3.24 (1.651–6.352)	0.001*
**Smoking**				
No	135 (75.4%)	44 (24.6%)	1	
Yes	52(91.2%)	5 (8.8%)	1.22 (1.045–1.424)	0.010*
**AUDIT Score (Alcohol use)**				
Not taking alcohol	142 (75.9%)	45 (24.1%)	1	
Low risk	28 (90.3%)	3 (9.7%)	2.96 (0.859–10.190)	0.086*
Medium risk	16 ((94.1%)	1 (5.9%)	5.07 (0.654–39.305)	0.120*
Alcohol addiction	1	0		
**Physical Exercise**				
Yes	38 (23.8%)	122 (76.2%)	1	
No	11 (14.5%)	65 (85.5%)	1.84 (0.882–3.840)	0.101*
**BMI**				
Normal	102 (73.4%)	37 (26.6%)	1	
Underweight	12 (92.3%)	1 (7.7%)	4.35 (0.547–34.648)	0.165
Overweight	57 (85.1%)	10 (14.9%)	2.07(0.957–4.466)	0.064*
Obese	16 (94.1%)	1 (5.9%)	5.80(0.74345.310)	0.094*
**SW Monofilament**				
Normal	136 (%)	45 (%)	1	
Abnormal	51 (92.7%)	4 (7.3%)	4.219 (1.444–12.329)	0.008*

*bmi* body mass index, *cor* crude odds ratio, *ci* confidence interval, *dm* diabetes mellitus, *tb* tuberculosis

*P ≤ 0.2

**Table 6:** Multivariable logistic regression of factors associated with erectile dysfunction among diabetic men at KIUTH, FPRRH and HRRH

Variable	Erectile Dysfunction		cOR (95% CI)	*p*-value	aOR	*p*-value
	Yes	No				
Age (in Years)						
Less than 30	7	8	1			
30 to 39	9	11	0.935 (0.244–3.584)	0.922	0.786 (0.159–3.874)	0.767
40 to 49	29	12	2.762 (0.818–9.329)	0.102	2.967 (0.542–6.239)	0.210
50 to 59	51	7	8.327 (2.302–30.119)	0.001	10.620 (1.593–70.808)	0.015*
60 and above	91	11	9.455 (2.871–31.134)	0.000	7.221 (1.145–45.521)	0.0358*
**DM duration**						
less than 5 years	58	17	1		1	
5 to 10years	51	19	0.79 (0.370–1.674)	0.533	0.447 (0.165–1.211)	0.113
more than 10 years	78	13	1.76	0.166	0.689 (0.221–2.150)	0.521
**Fasting blood sugar**						
Normal (≤ 7%)	48	24	1			
Abnormal (>7%)	139	25	2.78 (1.453–5.321)	0.002	2.736 (1.187–6.303)	0.018*
**HbA1c (%)**						
Normal (≤ 7%)	42	20	1			
Abnormal (>7%)	145	29	2.38 (1.224–4.631)	0.009	2.469 (1.057–5.766)	0.037*
**Hypertension**						
No	110	77	1			
Yes	15	34	3.24 (1.651–6.352)	0.001	1.032 (0.415 − 0.563)	0.946
**Smoking**						
No	135	44	1			
Yes	52	5	1.22 (1.045–1.424)	0.010	2.017 (0.601–6.766)	0.256
**AUDIT Score (Alcohol use)**						
Not taking alcohol	142	45	1			
Low risk	28	3	2.96 (0.859–10.190)	0.086	1.688 (0.410–6.947)	0.468
Medium risk	16	1	5.07(0.654–39.305)	0.120	2.214 (0.251–19.565)	0.475
Alcohol addiction	1	0				
**Physical Exercise**						
Yes	38	122	1			
No	11	65	1.84 (0.882–3.840)	0.101	1.069 (0.419–2.726)	0.888
**BMI**						
Normal	102	37	1			
Underweight	12	1	4.35 (0.547–34.648)	0.165	9.573 (0.790–11.596)	0.076
Overweight	57	10	2.07(0.957–4.466)	0.064	0.789–4.710)	0.150
Obese	16	1	5.80 (0.743–45.310)	0.094	15.183 (1.168–19.739)	0.038*
**SW Monofilament**						
Normal	136	45				
Abnormal	51	4	4.23 (1.444–12.325)	0.008	1.005 (0.281–3.593)	0.994

*BMI *Body Mass Index,* coR *crude odds ratio,* aOR *adjusted Odds ratio,* CI *Confidence interval,* DM *Diabetes Mellitus,* TB *Tuberculosis

P*˂0.05

At the multivariable level; Patients who had HbA1c of greater than 7% were 2.47 times more likely to have erectile dysfunction than those with normal (aOR = 2.469 95%CI: 1.057–5.766, *p* = 0.037).

Patients who had Fasting Blood Sugar of greater than 7mmol/L were 2.7 times more likely to have erectile dysfunction than those with ≤ 7mmol/L (aOR = 2.736 95%CI:1.1187–6.303, *p* = 0.018).

Patients aged 40 to 49 years were 2.97 more likely to have erectile dysfunction than those aged less than 30 years (aOR = 2.967 95% CI: 0.542–6.239, *p* = 0.210). The odds were 10.62 times higher among those aged 50 t0 59 years (aOR = 10.620 95%CI: 1.593–70.808, *P* = 0.015) (Table 6).

Participants with obesity were 15.18 times more likely to have erectile dysfunction than those with normal BMI (aOR − 15.1833 95%CI: 1.168–19.739).

## Discussion

In this study, the mean age of the study participants was 55 years. 174 men (73.7%) had HbA1c greater than 7% while 164 men (69.5%) had a fasting blood glucose greater than 7mmol/L, implying that the majority of the study participants had a poorly controlled blood sugar.

187 men, equivalent to 79.2% in this study, had erectile dysfunction. This occurrence of erectile dysfunction in about 4 in 5 men with diabetes is a significantly high magnitude. The high prevalence may be because majority of study participants (73.7%) had inadequately controlled blood sugar These findings are almost similar to those found in studies from North West, Harar and Hawassa - Ethiopia and Sudan (85.5%, 83.8, 72.8% and 81.1% respectively) [26, 30, 31]. Similar findings were noted in one meta-analysis, which aimed at computing a pooled prevalence of erectile dysfunction among diabetic patients in African countries [11].

Our results were different from a single-centre cross-sectional study in Nigeria, which had a prevalence of 58.9% [32]. This difference may be attributed to the methodological differences. The study in Nigeria was a single-centre study and included only men in the age bracket of 30–80 years. Another study in Nigeria recorded a relatively very high prevalence of 94.7% [33]. The difference may be since the former study assessed Erectile dysfunction among Type 2 diabetes, unlike the current study, which did not specify the type of diabetes. The type 2 diabetes patients are more prone to get more vascular complications compared to type 1 [34]. A pooled prevalence of sexual dysfunction in one Systematic review and meta-analysis for global data found a prevalence of 35.4% in the American region. This lower figure may be because of highly advanced diabetic care and more access to health care in America than in our setting [12].

The study conducted at Muhimbili National Referral Hospital in Tanzania found a contrasting prevalence of sexual dysfunction (55.1%) [15]. The difference may be attributed to the IIEF-15 questionnaire used in the former study and the IIEF-5 used in the current study. A systematic review on the global burden of ED among DM presented that the global pooled prevalence of ED was 65.8% and for Africa was 62.9% [35]. This finding is relatively less than the magnitude in our study, which may be attributed to the methodological design; the former being a systematic review and the latter being a cross-sectional study.

The majority of patients in this study had Mild to moderate erectile dysfunction (36.4%), with a minority having severe ED. 21.9%, 22.4% and 19.3% were for mild, moderate and severe erectile dysfunction, respectively. These findings are comparable to those of the study in Eastern Sudan, which showed that 24.3% had severe ED [30].

Our findings are also relatively similar to those of the hospital-based study in Ethiopia, which found that the majority of the patients had moderate erectile dysfunction (37.5%) [26]. In the other two studies in Ethiopia majority of patients had mild and moderate erectile dysfunction [9, 20]. In the same two studies, had very few participants with severe erectile dysfunction; 2.8% and 5.2% respectively. The difference may be due to variation in openness about sexual life and baseline socio-demographic characteristics. The two studies only had 30.4 and 18.5% being older than 60 years of age respectively, while our study had 43.2% of participants older than 60 years.

The study in Tanzania showed contrasting results from ours. The study found that about half of the diabetic patients with erectile dysfunction had severe erectile dysfunction [15]. This inconsistency may be related to a difference in the data collection tool used. The former used the 15 questions (IIEF-15) while the current study used a 5-question assessment (IIEF-5).

The present study found that age was an independent factor for the development of ED among diabetic men. Men aged above 60 years were 6.14 times more likely to get ED than those of 18-30years. Those aged 31–45 and 46–60 years were 2.42 times and 5.28 times, respectively, more likely to develop ED than those with 18–30 years of age. These findings are in agreement with those at Muhimbili National Referral Hospital in Tanzania, which found that increasing age poses an increased risk for ED; 30–44 (3.99 times), 45–59 (7.02 times), and 60+ (15.73 times) compared to those aged less than 30 years of age [15]. The study in Eastern Ethiopia and in Southern Ethiopia also found corresponding results, depicting that the odds of suffering from ED increase with the increase in age [20, 31]. The present study had findings aligned in the same line with those of a systematic review and meta-analysis of ED among DM patients in Africa by which found that those aged above 40 years have a 24% increased likelihood of ED than younger patients [11].

In the current study, fasting blood glucose (FBS) was significantly associated with ED. Patients with FBS greater than 7.0mmol/L were 2.50 times more likely to have ED than those with the FBS of less than or equal to 7.0mmol/L (aOR = 2.50, 95% CI: 1.106–5.657, *p* = 0.028). These results are consistent with the results of other cross-sectional studies done in Tanzania [15] and Ethiopia [9]. Findings similar to the present study were also reported by the study conducted in Ethiopia found that an FBS greater than 7mmol/L increases the risk of ED by 10 times [20].

Poor glycaemic control, evidenced by HbA1c of greater than 7% was associated with a high prevalence of ED in this study. This level of HbA1c was associated with an increased likelihood of occurrence of ED by 2.44 times. This echoes the findings of the study in Eastern Sudan by [30] which concluded that patients with uncontrolled DM had 3.38 times the odds of developing ED. Similar findings were noted in studies in Tanzania and Ethiopia [15, 31]. Our findings are also in keeping with the conclusion of the meta-analysis of several studies done in Africa on risk factors of ED in DM, which found that HbA1c less than 7% was a protective factor for ED [11]. Another meta-analysis and systematic review on risk factors of ED in Diabetic men re-echoed a similar finding that higher HbA1c had odds of 1.44 of leading to ED [36].

The current study also revealed that patients who were obese were 15.18 times more likely to have erectile dysfunction than those who had a normal Body mass index (aOR = 15.183, 95%CI:1.168–19.739, *p* = 0.038). This was in contrast to studies done in Sudan [30], in Tanzania [15] and Ethiopia [20]. The difference may be because the current study recruited patients from three different centres, while the former were single-centre studies.

The findings of the present study are in agreement with a systematic review and meta-analysis of African studies by [11] which concluded that BMI was associated with erectile dysfunction.

### Study strengths and limitations

This was the first multicenter-based study to assess erectile function among DM patients in Uganda. The study provides a broader spectrum and view of the magnitude of the ailment in the Western region. The study limitations included: Serum testosterone levels were not measured, other vascular complications were not studied and the study did not differentiate the occurrence of ED in type 1 versus type 2 diabetes mellitus.

## Conclusion

Erectile dysfunction is a real problem among men with diabetes mellitus. The overall prevalence of erectile dysfunction among diabetic men at KIUTH, FPRRH and HRRH highlights its widespread burden among diabetic men. The figures depicted that about 4 in 5 diabetic men had erectile dysfunction, of which no significant variation was seen from one sub-region to another. Increasing age and poor glycemic control play a significant role in putting men at risk of erectile dysfunction. These findings underscore the importance of early screening and tailored management strategies to improve the quality of life for diabetic men at risk of ED.

Routine screening for Erectile Dysfunction among men with diabetes mellitus may be a cornerstone for the early diagnosis. This is because most men may not directly talk about this sexual disorder when they come for routine evaluation. We recommend the timely addressing of the sugar control and openly asking men about their erectile functioning by health workers in the diabetes clinics. This may aid early diagnosis and, therefore, early intervention and improvement in quality of life. Future studies should focus on causality, therapeutic interventions and qualitative data on the subject matter in more regions in the country.

## Supplementary Information

Supplementary material 1.

## Data Availability

Data will be available by requesting from the authors (venanceemmanuel4@gmail.com, OR via venance@kiu.ac.ug).

## Abbreviations

ADA: American diabetes association
AUA: American urological association
AIDS: Acquired immunodeficiency syndrome
ED: Erectile dysfunction
HbA1c: Glycated hemoglobin
IIEF 5: International index of erectile function
OR: Odds ratio
