# Supplementary material for: Erectile dysfunction among diabetic patients in Western Uganda: prevalence and associated factors in a multicentre study across three selected clinics
Source: BMC Endocr Disord. 2025 Oct 23;25:238. doi: 10.1186/s12902-025-02048-2 (PMC12548112; doi:10.1186/s12902-025-02048-2)
Supplement: Supplementary file 1 — Supplementary material 1. [file 12902_2025_2048_MOESM1_ESM.pdf]

Appendix: Investigator Administered Questionnaire (English version)

HOSPITAL CODE ……………………………………………………

Study Number: |___|___|___|___|

1	Date of interview:	|___|___|/|___|___|/|___|___|___|___| (dd/mm/yyyy)	
**A. SOCIO - DEMOGRAPHIC DATA**			
2	What is your age?<br><br>|___|___| Completed years	3	What is your district of residence?<br><br>|___________________________________|
4	What is the residence type?<br><br>1= Rural 2 = Urban	5	What is your tribe?<br><br>1. Mutooro<br>2. Munyoro<br>3. Munyankore<br>4. Mukiga<br>5. Mufumbira<br>6. Others (Specify…………………………….)
6	What is your primary occupation?<br><br>1= Unemployed<br><br>2= Business<br><br>3= Peasant Farmer<br><br>4= Student	7	What is your level of education?<br><br>1= non formal<br><br>2= Primary education<br><br>3= Secondary education<br><br>4=Tertiary education

<table>
<tr><td></td><td colspan="4">5= Professional
6= Manual
7= Self employed
8= Other (Specify…………………….)</td><td>8</td><td colspan="4">What is your marital status?
1= Single
2= Married/Cohabiting
3= Separated/divorced
4= Widows</td></tr>
<tr><td colspan="10">B. MEDICAL HISTORY</td></tr>
<tr><td>9</td><td colspan="4">Duration since diabetes mellitus diagnosis
|___|___| years OR ……..months (if less than a year)</td><td>10</td><td colspan="4">Duration on taking drugs of Diabetes
|___|___| years OR ……months (if less than a year)</td></tr>
<tr><td>11</td><td colspan="4">Are you suffering from any of these conditions (Tick the box)</td><td>12</td><td colspan="2">Are you on any treatment?</td><td>13</td><td>Duration on treatment in months
(if applicable)</td></tr>
<tr><td></td><td></td><td>1=Yes</td><td>2=No</td><td>3=DK</td><td></td><td>1=Yes</td><td>2=
No</td><td></td><td></td></tr>
<tr><td>a</td><td>HIV</td><td>□</td><td>□</td><td>□</td><td>A</td><td>□</td><td>□</td><td>A</td><td>|___|___|___|___|</td></tr>
<tr><td>b</td><td>Hypertension</td><td>□</td><td>□</td><td>□</td><td>B</td><td>□</td><td>□</td><td>B</td><td>|___|___|___|___|</td></tr>
<tr><td>c</td><td>Tuberculosis</td><td>□</td><td>□</td><td>□</td><td>C</td><td>□</td><td>□</td><td>C</td><td>|___|___|___|___|</td></tr>
<tr><td>g</td><td>Other</td><td>□</td><td>□</td><td>□</td><td>D</td><td>□</td><td>□</td><td>D</td><td>|___|___|___|___|</td></tr>
<tr><td>h</td><td>If other, specify</td><td colspan="3">|______________________|</td><td></td><td></td><td></td><td></td><td></td></tr>
<tr><td>13</td><td colspan="4">How often do you attend diabetic clinic?</td><td>14</td><td colspan="4">If other, specify:</td></tr>
</table>

	1= Once per month<br>2= At least every 3 months<br>3= Never<br>4 = Other		____________________________
**C. BEHAVIOURAL FACTORS**			
15	Have you ever smoked cigarettes?<br>1 = Yes 0 = No	16	If ever smoked, how long have you been or did you smoke? |___|___|___|___| months
17	If ever smoked, are you currently smoking?<br>1 = Yes 0 = No	18	If stopped smoking, how long since you stopped?<br>|___|___| months
19	Number of cigarettes smoked per day<br>|___|___|	20	How many days a week do you smoke? |___|___| days
21	How often do you have a drink containing alcohol?<br>0 = Never 1 = monthly or less<br>2= 2-4 times a month 3= 2-3 times a week<br>4= 4 or more times a week	22	How often do you have 5 or more drinks in one occasion?<br>0= Never 1= less than monthly<br>2= monthly 3= weekly 4= Daily
23	How many drinks containing alcohol do you have on a typical day when you are drinking?	24	How often during the last year have you found that you were not able to stop drinking once you had started?

	0= Never 1= 3-4 2= 5-6 3= 7-9 4= 10 or more		0= Never 1= less than monthly<br><br>2= monthly 3= weekly 4= Daily
25	How often during the last year have you failed to do what was normally expected of you because of drinking?<br><br>0= Never 1= less than monthly<br><br>2= monthly 3= weekly 4= Daily	26	How often during the last year have you needed a first drink in the morning to get yourself going after a heavy drinking session?<br><br>0= Never 1= less than monthly<br><br>2= monthly 3= weekly 4= Daily
27	How often during the last year have you had a feeling of guilt or remorse after drinking?<br><br>0= Never 1= less than monthly<br><br>2= monthly 3= weekly 4= Daily	28	How often during the last year have you been unable to remember what happened the night before because of your drinking?<br><br>0= Never 1= less than monthly<br><br>2= monthly 3= weekly 4= Daily
29	Have you or someone else been injured because of your drinking?<br><br>0= No 2= Yes, but not in the last year 4= Yes, during the last year	30	Has relative, friend, doctor, or other health care worker been concerned about your drinking or suggested you cut down?<br><br>0= No 2= Yes, but not in the last year 4= Yes, during the last year
31	Do you engage in physical exercise?		
32	How often do you do physical exercise?	33	How long do you spend doing physical exercise?

<table>
<tr><td></td><td>1= Once a week 2= Twice a week
3= Thrice times a week 4= 4 times a week
5= At least five times a week</td><td></td><td colspan="2">1= Less than 30 mins 2= 30-45 mins
3=45mins or more</td></tr>
<tr><td colspan="5">D. MEDICAL FACTORS</td></tr>
<tr><td>34</td><td>Type of diabetic therapy</td><td>1=Yes</td><td>0= No</td><td></td></tr>
<tr><td>A</td><td>Oral hypoglycemics</td><td>☐</td><td>☐</td><td></td></tr>
<tr><td>B</td><td>Insulin therapy</td><td>☐</td><td>☐</td><td></td></tr>
<tr><td>C</td><td>Other</td><td>☐</td><td>☐</td><td></td></tr>
<tr><td>D</td><td>If other, specify</td><td colspan="3">|________________________________</td></tr>
<tr><td></td><td colspan="4">Examination findings</td></tr>
<tr><td>35</td><td>Height: |___|___|:|___| meters</td><td>36</td><td colspan="2">Weight: |___|___|:|___| kg</td></tr>
<tr><td>37</td><td>BMI(in Kg/m^2):
1.< 18.5 (underweight)
2. 18.5-24.9 (healthy weight)
3. 25.0-29.9 (obesity class I)
3. 30.0-34.9 (obesity class II)
4. >= 40 (extreme obesity)</td><td>38</td><td colspan="2">Monofilament assessment test score
a) Normal
b) Abnormal</td></tr>
<tr><td>39</td><td>Systolic blood pressure: |___|___|___| mmHg</td><td>40</td><td colspan="2">Diastolic blood pressure: |___|___|___| mmHg</td></tr>
<tr><td></td><td>General Laboratory findings</td><td></td><td></td><td></td></tr>
<tr><td>41</td><td>Fasting blood sugar: |_______________|</td><td>42</td><td colspan="2">Glycosylated Hb: |________________|</td></tr>
</table>

Alcohol Use Disorders Identification Test (AUDIT), WHO, 2019

1. How often do you have a drink containing alcohol?

(0) Never (Skip to Questions 9-10)
(1) Monthly or less
(2) 2 to 4 times a month
(3) 2 to 3 times a week
(4) 4 or more times a week

2. How many drinks containing alcohol do you have on a typical day when you are drinking?

(0) 1 or 2
(1) 3 or 4
(2) 5 or 6
(3) 7, 8, or 9
(4) 10 or more

3. How often do you have six or more drinks on one occasion?

(0) Never
(1) Less than monthly
(2) Monthly
(3) Weekly
(4) Daily or almost daily

4. How often during the last year have you found that you were not able to stop drinking once you had started?

(0) Never
(1) Less than monthly
(2) Monthly
(3) Weekly
(4) Daily or almost daily

5. How often during the last year have you failed to do what was normally expected from you because of drinking?

(0) Never
(1) Less than monthly
(2) Monthly
(3) Weekly
(4) Daily or almost daily

6. How often during the last year have you been unable to remember what happened the night before because you had been drinking?

(0) Never
(1) Less than monthly
(2) Monthly
(3) Weekly
(4) Daily or almost daily

7. How often during the last year have you needed an alcoholic drink first thing in the morning to get yourself going after a night of heavy drinking?

(0) Never
(1) Less than monthly
(2) Monthly
(3) Weekly
(4) Daily or almost daily

8. How often during the last year have you had a feeling of guilt or remorse after drinking?

(0) Never
(1) Less than monthly
(2) Monthly
(3) Weekly
(4) Daily or almost daily

9. Have you or someone else been injured as a result of your drinking?

(0) No
(2) Yes, but not in the last year
(4) Yes, during the last year

10. Has a relative, friend, doctor, or another health professional expressed concern about your drinking or suggested you cut down?

(0) No
(2) Yes, but not in the last year
(4) Yes, during the last year

Total AUDIT Score………………………………..

The test contains 10 multiple choice questions on quantity and frequency of alcohol consumption, drinking behaviour, and alcohol-related problems or reactions.

The answers are scored on a point system; a score of more than eight indicates an alcohol problem.

The International Index of Erectile Function (IIEF-5) Questionnaire (American Urological Association, 2018)

S/N	Over the past 6 months	1	2	3	4	5
1	How do you rate your confidence that you could get and maintain an erection?	Very low	Low	Moderate	High	Almost always/\Always
2	When you had erections with sexual stimulation, how often were your erections hard enough for penetration?	Never/Almost never	A few times (Much less than half the times)	Sometimes (About half the time)	Most times (Much more than half the time)	Always/Almost always
3	. During sexual intercourse, how often were you able to maintain your erection after you had penetrated (entered) your partner	Almost never/never	A few times (much less than half the time)	Sometimes (about half the time)	Most times (much more than half the time)	Almost always/ always
4	During sexual intercourse, how difficult was it to maintain your erection to completion of intercourse?	Extremely difficult	Very difficult	Difficult	Slightly difficult	Not difficult
5	When you attempted sexual intercourse, how often was it satisfactory for you?	Almost never/never	A few times (much less than half the time)	Sometimes (about half the time)	Most times (much more than half the time	Almost always/ always

IIEF-5 scoring:

The IIEF-5 score is the sum of the ordinal responses to the 5 items.

22-25: No erectile dysfunction

17-21: Mild erectile dysfunction

12-16: Mild to moderate erectile dysfunction

8-11: Moderate erectile dysfunction

5-7: Severe erectile dysfunction

Total Score _________
